# A systematic review of the diagnostic accuracy of Loop-mediated-isothermal AMPlification (LAMP) in the diagnosis of invasive meningococcal disease in children

**DOI:** 10.1186/s12887-019-1403-0

**Published:** 2019-02-07

**Authors:** Thomas Waterfield, Derek Fairley, Bronagh Blackwood, James McKenna, Michael D. Shields

**Affiliations:** 1 Centre for Experimental Medicine, Queen’s University Belfast, Wellcome Wolfson Institute of Experimental Medicine, 97 Lisburn Road, Belfast, BT9 7AE UK; 2 Belfast Health and Social Care Trust, Belfast, UK

**Keywords:** Meningococcal, Meningitis, Sepsis, *Neisseria meningitidis*, Test accuracy, Meta-analysis

## Abstract

**Background:**

The early recognition of meningococcal disease in children is vital. During the prodrome however, meningococcal infection presents similarly to many self-limiting viral infections. This mandates a cautious approach with many children receiving unnecessary broad-spectrum parenteral antibiotics. Advances in nucleic acid amplification techniques mean that it is now possible to test for *Neisseria meningitidis* DNA using Loop-mediated-isothermal AMPlification (LAMP). This technique is quicker than traditional PCR techniques and can be performed using simple equipment.

**Methods:**

Prior to performing this systematic review, a protocol was developed adhering to PRISMA P standards and underwent full external peer review. This systematic review was registered with PROSPERO (CRD42017078026). The index test assessed was LAMP for *Neisseria meningitidis* and the reference standard was culture or qPCR of a sterile site detecting *Neisseria meningitidis.*

**Results:**

We identified 95 records in total: 94 records from the electronic databases and 1 additional study from the grey literature. After removal of duplicates, 36 studies were screened, and 31 studies excluded based on the title/abstract. Five full text studies underwent full text review and three studies, including 2243 tests on 1989 patients aged between 7 days and 18 years were included in the final systematic review. In all studies the LAMP assay and qPCR primers were directed against the ctrA region of the *Neisseria meningitidis* bacteria. The diagnostic accuracy of LAMP testing for invasive meningococcal disease was reported as high (sensitivity 0.84–1.0 and specificity 0.94–1.0) in all studies irrespective of the sample tested (CSF, Blood, Swab).

**Conclusions:**

We included three studies with 2243 tests on 1989 patients using CSF, blood samples or naso/oropharyngeal swabs. The studies were all of a high quality and deemed at low risk of bias. Results show that LAMP testing on blood and CSF was highly accurate when compared to qPCR/culture.

LAMP testing for *Neisseria meningitidis* is fast and highly accurate and therefore ha*s* the potential to be used to rapidly rule in/out meningococcal disease in children. Given the life-threatening nature of meningococcal infection further research is required to demonstrate the safety and efficacy of using LAMP testing for *Neisseria meningitidis* as a rule in/out test.

**Trial registration:**

This systematic review was registered prospectively with PROSPERO on the 29/11/2017 (CRD42017078026).

**Electronic supplementary material:**

The online version of this article (10.1186/s12887-019-1403-0) contains supplementary material, which is available to authorized users.

## Background

Despite successful vaccination programmes meningococcal disease (MD) remains a leading infectious cause of septicaemia and death in children worldwide [1–5]. The early diagnosis of MD significantly improves outcomes with reduced morbidity and mortality. The challenge is however, that during the prodrome invasive MD is indistinguishable from many self-limiting viral infections [4–6]. This mandates a cautious approach to the management of these children with many receiving parenteral antibiotics pending culture results [7]. Despite this approach children are still being diagnosed late due to the difficulties in identifying children who have MD as opposed to a simple viral illness [4, 7], while many more are being treated “just in case”.

Currently there is no biomarker, or combination of biomarkers, with sufficient diagnostic accuracy to be used as rule in/ rule out tests for invasive MD in children [8–13]. Attention has therefore moved towards faster and easier molecular testing to allow for earlier diagnosis. This has several potential benefits (i) rapid diagnosis of invasive MD at presentation could help to tailor initial treatment (ii) rapid exclusion of invasive MD could shorten the course of parental antibiotics, facilitate earlier discharge or appropriately direct the clinician’s attention towards other infectious diseases.

Rapid molecular testing exists in the form of Loop-mediated-isothermal AMPlification (LAMP) for *Neisseria meningitidis* [14–16]. The LAMP is a form of nucleic acid amplification that utilises specific looped primers and strand displacing DNA polymerase. LAMP has several advantages over traditional PCR techniques including (i) quicker testing, typically performed in under an hour (ii) it requires simpler equipment (iii) compared to PCR, LAMP is highly tolerant of biological fluids facilitating direct testing of clinical material [15–20]. It may therefore be possible to move molecular testing from centralised laboratories to clinical areas thereby significantly reducing time to diagnosis.

A systematic review is required to inform on the diagnostic accuracy of meningococcal LAMP in the paediatric population. Data from this systematic review will be useful in the development of clinical practice guidelines and for policy makers.

The aim of this systematic review was to determine the diagnostic accuracy of meningococcal LAMP in predicting and diagnosing invasive MD - defined as the identification of *Neisseria meningitidis* from a sterile site, blood or Cerebrospinal Fluid (CSF), using either Real-time PCR (e.g.TaqMan® PCR) or bacterial culture. In children less than 18 years of age.

## Methods

Prior to conducting this systematic review a protocol was produced in adherence to the standards of the Preferred Reporting Items for Systematic Reviews and Meta-Analyses (PRISMA) and registered prospectively on the 29/11/2017 with the International Prospective Register of Systematic Reviews (PROSPERO) - registration number CRD42017078026 [21, 22]. The protocol has undergone external peer review and was published in 2018 [23]. We used the Cochrane recommendations for reporting systematic reviews and meta-analysis of diagnostic accuracy studies [24].

### Eligibility criteria

We included all prospective, retrospective and randomised controlled trials that assessed the performance of LAMP in children (< 18 years of age) with potential invasive meningococcal disease. For the purpose of this review the index test was defined as LAMP testing for *Neisseria meningitidis*. Index testing could have been performed using blood, cerebrospinal fluid and naso/oropharyngeal swabs. Commercial and laboratory developed tests were eligible. The reference standard was identification of *Neisseria meningitidis* from a sterile site (blood or CSF) using either bacterial culture or real-time PCR.

### Why include naso/oropharyngeal swabs?

Naso/oropharyngeal swabs are minimally invasive and easy to collect in young children when compared with blood and CSF samples. Given that *Neisseria meningitidis* typically invades through the naso/oral mucosa it may be possible to tests naso/oropharyngeal swabs to predict those children with early invasive meningococcal infection [25]. The potential disadvantage of this approach however, is that detection of harmless carriage may reduce the specificity of this approach. Carriage rates of capsular strains of *Neisseria meningitidis* are however, typically low in early childhood increasing to a peak in adolescence [26, 27]. It may therefore be possible to use naso/oropharyngeal swab testing as an early and reliable predictor of disease in young children [26].

### Information sources and search strategies

An electronic search strategy was developed in collaboration with the Queen’s University Belfast Medical Librarian (RF). We searched MEDLINE, Embase, Web of Science, Scopus and the Cochrane Library inclusive of Cochrane Controlled Trials Register from inception to 10th May 2018. We did not apply language restrictions. The Medline search strategy is attached as a Additional file 1. In addition, we contacted the manufacturers of commercially available meningococcal LAMP tests and searched conference abstracts.

### Study selection and data extraction

Two reviewers (TW, MDS) independently screened all abstracts and titles against inclusion criteria and assessed full text publications for eligibility. The same two reviewers independently judged study quality using the Quality Assessment of Diagnostic Accuracy Studies (QUADAS-2) tool [28]. Disagreements were resolved by consensus or arbitration by a third party (DF).

Using a pre-piloted data extraction tool (see Additional file 2), two reviewers (TW, MDS) independently extracted the following information:

- *Study characteristics*: author, year of publication, country, design, sample size, clinical setting, number studied, number of drop-outs with reason, and funding source.
- *Population characteristics*: inclusion/exclusion criteria; patient demographics
- *LAMP Testing*: timing of sampling; method of sampling (e.g naso/oropharyngeal swab, blood, CSF)
- *Gold standard*: Real-time PCR (e.g.TaqMan® PCR) or sterile site bacterial culture (i.e blood/CSF)
- *Outcomes*: True positives, false positives, true negatives, and false negatives were extracted to construct a diagnostic contingency (2-by-2) table.

### Data analysis

Statistical analysis and data synthesis were performed by TW and LAMP test result data were compared to the reference test. The true positive, true negative, false positive and false negative rate were recorded and used to create a 2 × 2 tables. From these tables inferred statistics were calculated including sensitivity and specificity with 95% confidence intervals. Meta-analysis to provide pooled sensitivity and specificity data were not performed due to the small number of studies available. All analysis was performed using *Review Manager (RevMan) Version 5.3. Copenhagen: The Nordic Cochrane Centre, The Cochrane Collaboration, 2014.*

## Results

### Study inclusion

**Fig. 1:**
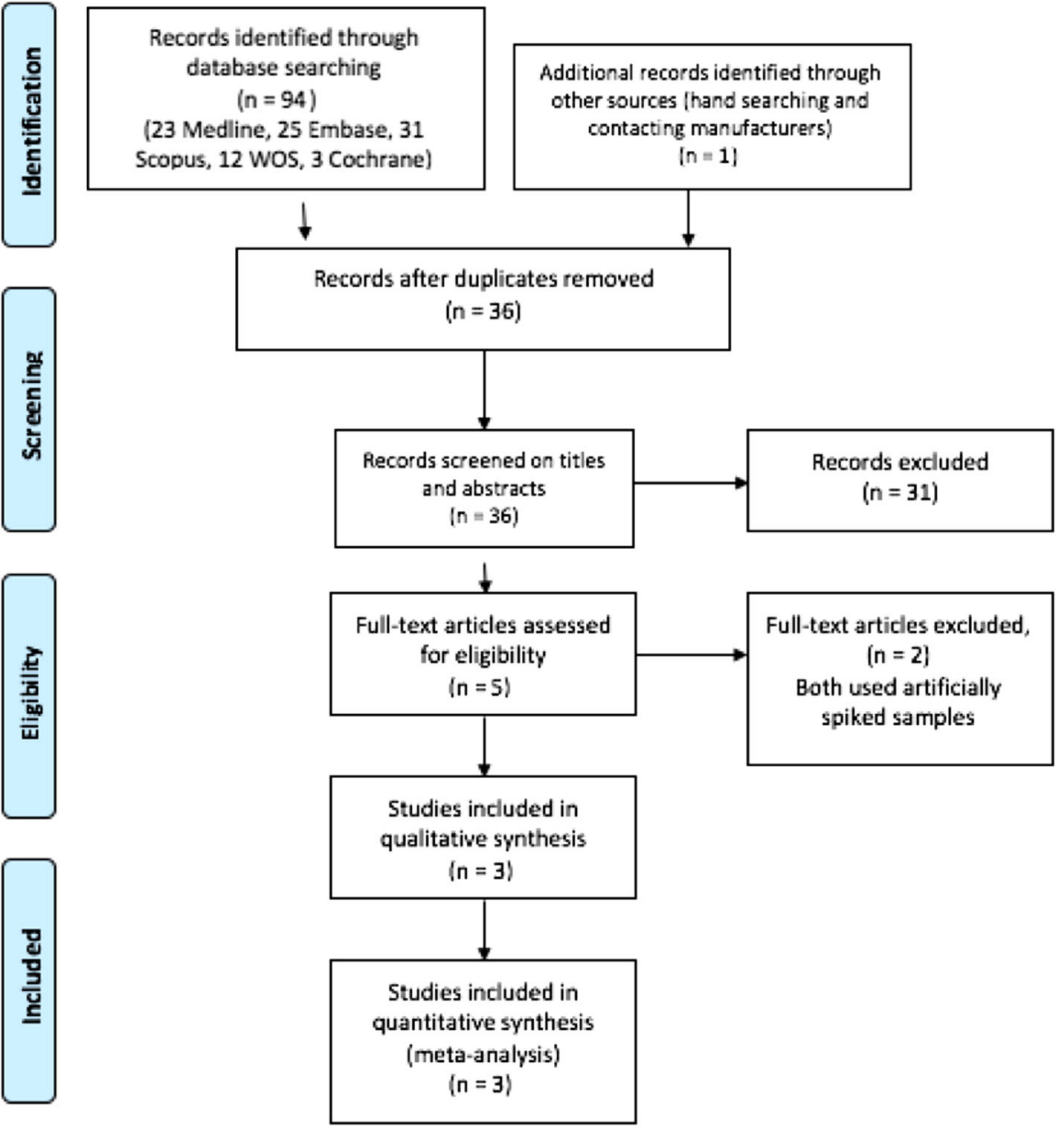
PRISMA Flow Diagram

We identified 95 records in total: 94 records from the electronic databases and 1 additional study from the grey literature. After removal of duplicates, 36 studies were screened, and 31 studies excluded based on the title/abstract. All of the 31 studies screened and excluded were not relevant to the systematic review. Five full text studies underwent full text review, and three studies were included in the final systematic review (see Fig. 1). The two studies excluded both used “spiked specimens” as opposed to true clinical specimens [19, 29]. Spiked specimens involve the adding of *Neisseria meningitidis* DNA to human specimens as opposed to detecting wild type *Neisseria meningitidis* in disease.

### Study characteristics and risk of bias

Three studies including 2243 tests on 1989 patients aged between 7 days and 18 years were included [14–16]. All three studies were cohort studies, two were conducted in the United Kingdom and one in Vietnam, China and Korea [14–16]. In all studies the LAMP assay and qPCR primers were directed against the ctrA region of the *Neisseria meningitidis* bacteria. The *ctrA* gene sequence is genetically conserved across all pathogenic (capsular) strains of the *Neisseria meningitidis* bacteria. Study characteristics are outlined in Table 1. In total there were 1595 tests using CSF samples, 345 using blood samples and 396 using naso/oropharyngeal swabs.

**Table 1:** Study Characteristics

Author	Year Published	Number of Patients	Country	Design	Clinical Setting	Funding	Drop Outs	Inclusion Criteria	Exclusion Criteria	Age Range	LAMP Specimens	Timing of sampling	Reference Standard
Lee (14)	2015	1574	Vietnam, China & Korea	Retrospective cohort study	Suspected Meningitis	State Funded	0	Suspected Meningitis	> 5 years of age	< 5 years	CSF	Retrospective	qPCR
Bourke (15)	2015	148	UK	Prospective cohort study	Paediatric ED	State Funded	13 No consent	Suspected meningococcal infection	> 14 years of age	17 days to 12 years	CSF, Blood, Swab	Prospective	qPCR Blood Culture
McKenna(16)	2010	267	UK	Retrospective cohort study	Children’s Hospital	State Funded	11 Excluded (> 18 years of age)	Suspected meningococcal infection	None	7 days to 18 years	CSF Blood Swab	Retrospective	qPCR

*CSF* Cerebrospinal fluid, *qPCR* Quantitative PCR

The study by McKenna et al. was performed in the UK in both adults and children with a total 267 patients recruited. The age range was 7 days to 57 years and the median age was 1 year (16). Of the 267 patients 256 were children under 18 years of age. We contacted the corresponding author and obtained the dataset pertaining only to those participants under 18 years of age. These data included 256 separate patients with 203 individual blood samples (either serum or EDTA), 21 patients with CSF samples and 155 patients with naso/oropharyngeal swabs including “respiratory swabs”. The study by Bourke et al. included 148 patients aged 17 days to 12 years of age and was performed in the UK. Of the 148 patients 141 had naso/oropharyngeal swab results and 144 had blood results. There was also an analysis of 8 CSF samples of which 7 had both LAMP and qPCR results (15). The study by Lee et al. included 1574 patients under 5 years of age with suspected meningitis recruited from across Vietnam, China and Korea. All children underwent both LAMP and qPCR on CSF samples.

**Fig. 2:**
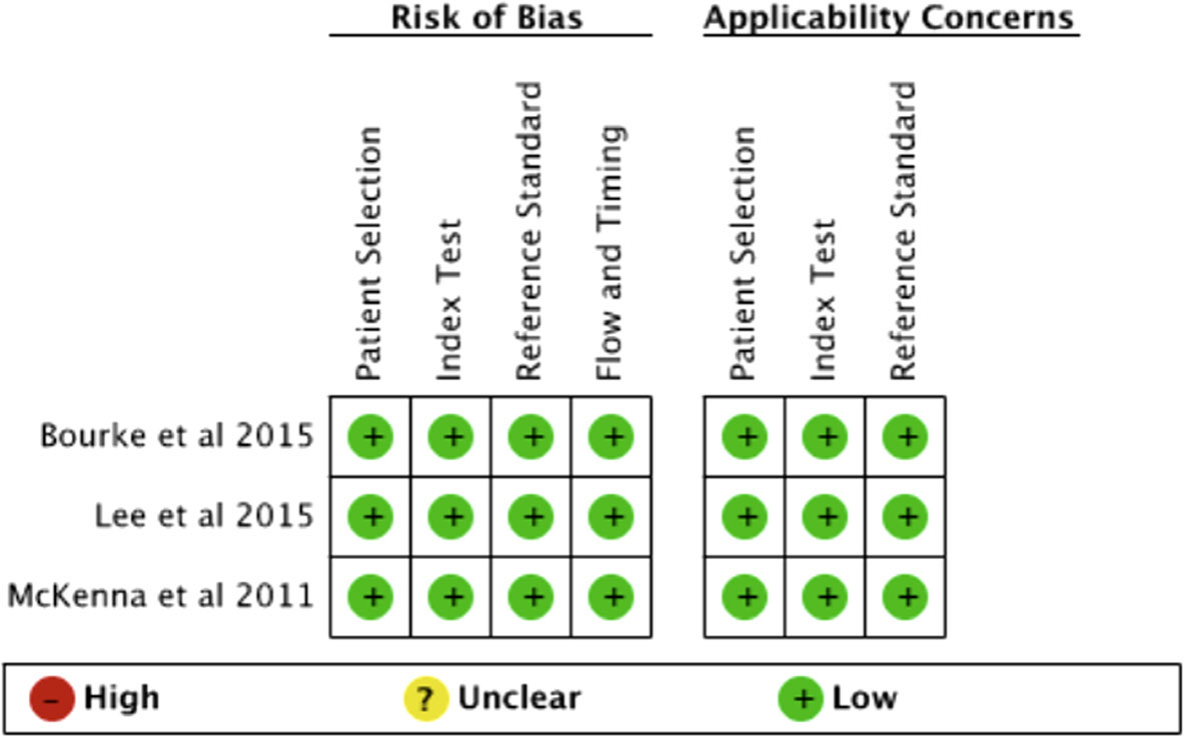
Risk of bias and applicability concerns summary

The methodological quality of the studies was judged as at low risk of bias (Fig. 2). In all instances the reference test was performed blinded to the result of the test being evaluated.

### Diagnostic accuracy

**Fig. 3:**
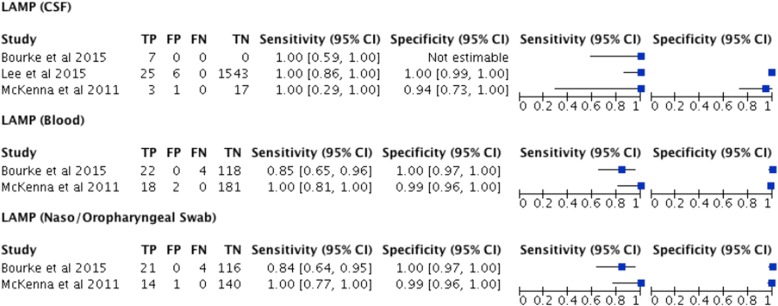
Reported diagnostic accuracy by study and test with forest plots

The diagnostic accuracy of LAMP testing for invasive meningococcal disease was reported as high (sensitivity 0.84–1.0 and specificity 0.94–1.0) in all studies irrespective of the sample tested (CSF, Blood, Swab) (See Fig. 3).

#### LAMP (blood)

Two of the studies (Bourke et al. and McKenna et al), with 345 patients combined, provided diagnostic accuracy on LAMP testing for *Neisseria meningitidis* on blood specimens (either EDTA or Serum) [15, 16]. The sensitivity was reported as high in both the Bourke et al. study 0.85(95%CI 0.65 to 0.96) and the McKenna et al. study1.00(95%CI 0.81 to 1.00). The specificity of LAMP testing for *Neisseria meningitidis* on blood was also reported as high in both the Bourke et al. study 1.00(95%CI 0.97 to 1.00) and the McKenna et al. study 0.99(95%CI 0.96 to 1.00) [15, 16]. The number of studies was too small to perform a meta-analysis and report a pooled sensitivity or specificity of blood LAMP testing for *Neisseria meningitidis.* These data are summarised in Fig. 3.

#### Lamp (CSF)

All three studies including 1602 patients provided diagnostic accuracy data on LAMP testing for *Neisseria meningitidis* on CSF specimens [14–16]*.* Of these 1574 came from a single study by Lee et al. [14]. The sensitivity and specificity was reported as high in the largest cohort reported by Lee et al. 1.00(95%CI 0.86 to 1.00) and 1.00(95%CI 0.99 to 1.00) respectively [14]. LAMP testing of CSF was also found to be highly sensitive in the other two smaller studies with Bourke et al. reporting the sensitivity as 1.00(0.59 to 1.00) and McKenna et al. reporting the sensitivity as 1.00(0.29 to 1.00) [15, 16]. The specificity of LAMP testing for *Neisseria meningitidis* on CSF specimens was reported as high in the study by McKenna et al. 0.94(95%CI 0.73 to 1.00). The number of studies was too small to perform a meta-analysis and report a pooled sensitivity or specificity of CSF LAMP testing for *Neisseria meningitidis.* These data are summarised in Fig. 3.

#### LAMP (naso/oropharyngeal swabs)

Two of the studies (Bourke et al. and McKenna et al), with 296 patients combined, provided diagnostic accuracy on LAMP testing for *Neisseria meningitidis* on naso/oropharyngeal swab specimens [15, 16]. The sensitivity was reported as high in both the Bourke et al. study 0.84(95% 0.64 to 0.95) and the McKenna et al. study 1.00(95%CI 0.77 to 1.00). The specificity of LAMP testing for *Neisseria meningitidis* on naso/oropharyngeal swab specimens was also reported as high in both the Bourke et al. study 1.00(95%CI 0.97 to 1.00)and the McKenna et al. study 0.99(95%CI 0.96 to 1.00) [15, 16]. The number of studies was too small to perform a meta-analysis and report a pooled sensitivity or specificity of blood LAMP testing for *Neisseria meningitidis.* These data are summarised in Fig. 3.

## Discussion

This review was designed to determine the accuracy of LAMP testing for *Neisseria meningitidis* when compared to the existing reference standard of either Real-time PCR (e.g.TaqMan® PCR) or bacterial culture in children less than 18 years of age.

We included three studies with 2243 tests on 1989 patients using CSF, blood samples or naso/oropharyngeal swabs. The studies were all of a high quality and deemed at low risk of bias. Results show that LAMP testing on blood and CSF was highly accurate when compared to qPCR/culture with a sensitivity ranging from 0.85 to 1.00 and a specificity ranging from 0.94 to 1.00.

Similarly testing of naso/oropharyngeal swabs was highly accurate for predicting those children with invasive meningococcal disease (Blood/CSF positive qPCR or bacterial culture) with a sensitivity ranging from 0.84 to 1.00 and a specificity reported at 1.00. This is likely due to a combination of factors including (i) the LAMP assays used were directed against the ctrA region thereby only detecting pathogenic strains of *Neisseria meningitidis* that are typically associated with invasive disease and (ii) low carriage rates of capsular *Neisseria meningitidis* in young children. These findings raise the possibility that in young children that LAMP testing of naso/oropharyngeal swabs for *Neisseria meningitidis* could be used as a non-invasive and rapid test to identify those as risk of invasive meningococcal disease.

Further prospective research is required to determine where in the diagnostic pathway *Neisseria meningitidis* LAMP testing could be used and which specimen type is ideal. LAMP testing can be performed in under one hour in most instances suggesting it could be used (i) prior to initiation of antibiotic therapy or (ii) to tailor antibiotic therapy. The potential benefits of earlier diagnosis or exclusion of invasive MD include (i) redirecting the clinical team to other potential diagnoses (ii) earlier tailoring or stopping of antibiotic therapy (iii) potential shorter periods of hospital admission (iv) improved anti-microbial stewardship.

If LAMP testing for *Neisseria meningitidis* is to be used as a rapid rule out test as suggested above, then further studies are required to demonstrate the safety of this approach. The overall sensitivity of LAMP testing is high 0.85 to 1.00 but the existing studies using blood and naso-oropharyngeal swab testing are small with wide confidence intervals. Given the life-threatening nature of meningococcal infection it is important that any use of LAMP as a rule out test has excellent sensitivity and can be shown to be safe in clinical practice.

### Limitations

This systematic review has a number of limitations. The number of diagnostic accuracy studies reporting on LAMP for *Neisseria meningitidis* remains small. With the majority of data available from a single study (14).

This systematic review may also overestimate the diagnostic accuracy of LAMP testing on naso/oropharyngeal swabs to predict invasive meningococcal disease. The majority of the children in the reviewed studies were under 5 years of age and as such the carriage rates of capsular *Neisseria meningitidis* will have been low. Further research is required to determine the diagnostic test accuracy of LAMP testing for *Neisseria meningitidis* on naso/oropharyngeal swabs from older children and adolescents where carriage rates of capsular *Neisseria meningitidis* are higher.

Finally, it is entirely possible that LAMP techniques are more sensitive than the existing reference standard of qPCR and/or bacterial culture techniques. With LAMP testing it is possible to detect fewer than 10 copies of bacterial DNA. If LAMP testing is more sensitive than the existing reference standard, then this review would underestimate the specificity of LAMP by falsely assuming that a LAMP positive, but reference standard negative test was a false positive result.

## Additional files

Additional file 1: Medline Search Strategy. (DOCX 97 kb)

Additional file 2: LAMP-SR (Data Extraction Tool). (DOCX 58 kb)

## Abbreviations

CRP: C-reactive protein
CSF: Cerebrospinal fluid
DNA: Deoxyribonucleic acid
LAMP: Loop-mediated-isothermal AMPlification
MD: Meningococcal disease
NPV: Negative predictive value
PCR: Polymerase chain reaction
PCT: Procalcitonin
POCT: Point of care testing
PPV: Positive predictive value
RCT: Randomised Control Trial
