# Supplementary material for: A systematic review of the diagnostic accuracy of Loop-mediated-isothermal AMPlification (LAMP) in the diagnosis of invasive meningococcal disease in children
Source: BMC Pediatr. 2019 Feb 7;19:49. doi: 10.1186/s12887-019-1403-0 (PMC6366060; doi:10.1186/s12887-019-1403-0)
Supplement: Supplementary file 2 — LAMP-SR (Data Extraction Tool). (DOCX 58 kb) [file 12887_2019_1403_MOESM2_ESM.docx]

LAMP-SR (Data Extraction Tool)

- Study characteristics:

| Author |  |
| --- | --- |
| Year |  |
| Countries |  |
| Design |  |
| Sample size |  |
| Clinical setting |  |
| Total number studied |  |
| Dropouts |  |
| Reasons for dropouts |  |
| Funding source |  |

- Population characteristics:

| Inclusion Criteria |  |
| --- | --- |
| Exclusion Criteria |  |
| Ages |  |
| Gender |  |

- LAMP Testing:

| Timing of sample –  i.e. prospective/retrospective |  |
| --- | --- |
| Method of sampling  CSF/Blood/Throat swab/Other |  |
| Time to result |  |
| Test setting i.e. Laboratory/ POCT |  |
| Gold standard (qPCR/Culture) |  |

- Outcomes:

| All LAMP | LAMP+ | LAMP- |
| --- | --- | --- |
| PCR/Culture + |  |  |
| PCR AND Culture -ve |  |  |

| Blood LAMP | LAMP+ | LAMP- |
| --- | --- | --- |
| PCR/Culture + |  |  |
| PCR AND Culture -ve |  |  |

| CSFLAMP | LAMP+ | LAMP- |
| --- | --- | --- |
| PCR/Culture + |  |  |
| PCR AND Culture -ve |  |  |

| Throat Swab LAMP | LAMP+ | LAMP- |
| --- | --- | --- |
| PCR/Culture + |  |  |
| PCR AND Culture -ve |  |  |

| Other LAMP | LAMP+ | LAMP- |
| --- | --- | --- |
| PCR/Culture + |  |  |
| PCR AND Culture -ve |  |  |
